# Shape-morphing living composites

**DOI:** 10.1126/sciadv.aax8582

**Published:** 2020-01-17

**Authors:** L. K. Rivera-Tarazona, V. D. Bhat, H. Kim, Z. T. Campbell, T. H. Ware

**Affiliations:** 1 Department of Bioengineering, The University of Texas at Dallas, Richardson, TX, USA.; 2 Department of Biological Sciences, The University of Texas at Dallas, Richardson, TX, USA.

## Abstract

This work establishes a means to exploit genetic networks to create living synthetic composites that change shape in response to specific biochemical or physical stimuli. Baker’s yeast embedded in a hydrogel forms a responsive material where cellular proliferation leads to a controllable increase in the composite volume of up to 400%. Genetic manipulation of the yeast enables composites where volume change on exposure to l-histidine is 14× higher than volume change when exposed to d-histidine or other amino acids. By encoding an optogenetic switch into the yeast, spatiotemporally controlled shape change is induced with pulses of dim blue light (2.7 mW/cm^2^). These living, shape-changing materials may enable sensors or medical devices that respond to highly specific cues found within a biological milieu.

## Teaser

Baker’s yeast embedded in a hydrogel enables the engineering of smart materials that change shape in response to specific cues.

## INTRODUCTION

Materials that change shape enable mechanical activity in devices, such as smart garments, sensors, microfluidics, or drug delivery platforms (*1*–*4*). In these devices, traditional actuators, like solenoids, are too large, heavy, or power intensive to be used. Shape change in synthetic polymers and gels can be triggered using temperature, electric fields, or chemicals (*5*–*8*). The specificity of the response is dictated, and limited, by the physical characteristics of the material (*9*). One approach to induce specificity in the physical characteristics of a hydrogel is to build polymer networks from biomacromolecules, such as DNA, allowing the detection of analytes that directly bind to these constituents (*10*). Binding of designed DNA sequences can induce 100-fold volumetric hydrogel expansion by successive extension of cross-links, using a DNA hybridization cascade. In the design of chemically responsive hydrogels, this mechanism is limited to detection of analytes capable of highly specific binding motifs. In living organisms, direct DNA binding is not the typical mechanism by which sensing occurs. Genetic information in cells encodes components that enable appropriate responses to a wide range of specific chemical and physical cues.

Composites that combine the tunable properties of synthetic materials and the responsive nature of living organisms represent a powerful strategy to imbue multifunctionality in a single material. Several living composites have been previously reported including self-healing concrete (*11*), ethanol-producing three-dimensional (3D)–printed hydrogels (*12*), gels that self-heal using photosynthesis (*13*), and wearable fluorescent biosensors (*14*). However, these living composites lack the ability to respond mechanically to environmental cues. One example of a mechanically active living composite is a bilayer of an elastomer and mammalian muscles that bends through contraction and relaxation of the muscle cells (*15*). However, muscle cells only thrive over a very narrow set of conditions, limiting the range of applications where these materials can be used. Notably, shape change in living organisms is not limited to contraction of muscles. Tissue morphogenesis in animals and plants is controlled, in part, by cellular proliferation (*16*, *17*). However, a strategy that harnesses proliferation of living cells to control the shape change of synthetic materials has yet to be reported.

**Fig. 1:**
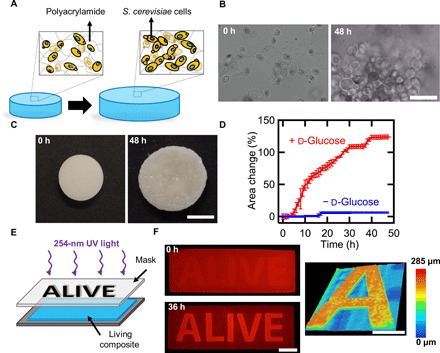
Controlled expansion of polyacrylamide gels by proliferation of yeast. (**A**) Schematic of shape change in living composites. In YPD, yeast proliferate and cause expansion in the polymer matrix. (**B**) Optical micrographs of a living composite before and after growth in medium. Scale bar, 30 μm. (**C**) Macroscopic expansion of a living composite gel with 6 wt % yeast. Scale bar, 7 mm. (**D**) Area change over time of a sample with 6 wt % yeast in the presence of medium with and without glucose. (**E**) Photopatterning process of a living composite. (**F**) Fluorescence images of a living composite after UV patterning (top) and after incubation in YPD (bottom). Scale bar, 10 mm. Topography of an initially flat living composite after exposure to YPD (right). Scale bar, 5 mm. Each data point represents the mean (*n* = 3), and error bars represent SD. Trend lines are only intended to guide the eye.

Here, we describe hybrid materials where living *Saccharomyces cerevisiae* (i.e., baker’s yeast or brewer’s yeast) embedded within a polyacrylamide hydrogel proliferates in response to a combination of environmental cues, which induces shape change in the composite (Fig. 1A). By controlling cell loading or hydrogel stiffness, we control the magnitude of volume change in the composites. This shape change is further controlled by patterning proliferation within a monolith. Critically, yeast provide a versatile platform for genetic engineering of the conditions required for proliferation. Using this control, we design composites that respond only in the presence of a single chirality of a single amino acid or to brief pulses of dim visible light. We harness this shape change to create microfluidic channels that respond selectively to fluids flowing through the channel.

## RESULTS AND DISCUSSION

*S. cerevisiae* is an ideal model organism to realize responsive, living composites. These unicellular organisms thrive within solid matrices (*12*), are much stiffer (1 to 10 MPa) (*18*) than many hydrogels (10 to 100 kPa) (*19*), and are known to survive over a wide range of conditions (*20*). Our key observation is that as these stiff cells proliferate within a solid hydrogel matrix, a global increase in volume is observed. We hypothesize that this volume increase is not due to ordinary swelling of a hydrogel, but instead is attributable to local displacement of the hydrogel by proliferating cells. After the composite is exposed to the appropriate conditions for cell growth, a marked increase in cell count can be observed (Fig. 1B). To quantify the effect of proliferation on macroscopic volume change, living composites were polymerized with pre-gel solution [0.9 billion to 1.1 billion cells/ml; 6 weight % (wt %) of dry yeast]. Composites were incubated in YPD (yeast extract, peptone, d-glucose) medium at 30°C for 48 hours. YPD contains the necessary nutrients for the yeast, and hence, cell proliferation–induced shape change occurs, resulting in a change in area of 124.2 ± 10.3% and a volume change of 200.9 ± 2.4% (Fig. 1C and movie S1). By incubating composites in medium without a fermentable carbon source (d-glucose), the area of the disk only increased by 6.3 ± 0.4%, as the yeast are incapable of proliferation (Fig. 1D and fig. S1). Similarly, hydrogels without encapsulated yeast incubated in YPD only undergo a volume change of 1.2 ± 0.5% (fig. S2). The shape change of the living composites in rich medium is also not attributable to passive cell size changes; cell viability is required for shape change to occur. We pattern cell viability, using ultraviolet (UV) light (254 nm) exposed through a mask, in living composites covalently bound to glass (Fig. 1E). Only the regions of the hydrogel not exposed to UV, the letters “ALIVE,” contain viable cells and undergo a volume increase on exposure to YPD. This expansion is greater than 110% of the initial film thickness after 36 hours (Fig. 1F and fig. S3). Shape change is accompanied by a change in topography, from smooth to rough, as the growing colonies deform the surface in a heterogeneous manner at the submillimeter scale. The described experiments show that yeast proliferation is the primary mechanism associated with volume change in these hybrid living materials.

**Fig. 2:**
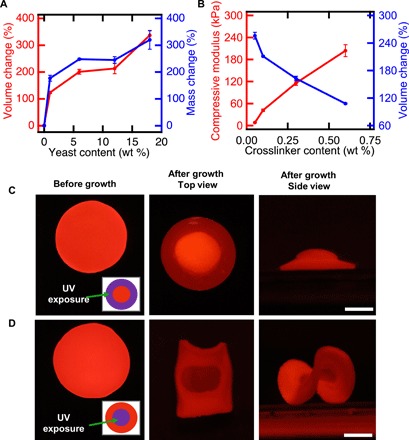
Shape change of living composites can be controlled. (**A**) Volume and mass change of living composites as a function of yeast content. (**B**) Compressive modulus and volume change as a function of cross-linker content. (**C**) Flat disk exposed to spatially patterned UV light (left) in a 3-mm-wide ring pattern (inset). After incubation in medium, a hat-like structure with positive Gaussian curvature is observed (center, right). Scale bar, 5 mm. (**D**) Flat disk exposed to spatially patterned UV light (left) in a 6-mm inner circle (inset). Upon incubation in medium, a saddle-like structure with negative Gaussian curvature is observed (center, right). Scale bar, 5 mm. Each data point represents the mean (*n* = 3), and error bars represent SD. Trend lines are only intended to guide the eye.

Proliferation-driven shape change can be tuned by controlling the initial composition of the living composite (Fig. 2, A and B). On varying the initial concentration of yeast from 1 to 18 wt %, the volume change after 48 hours increases from 123.8 ± 3.9% to 337.2 ± 17.4%. This volume change is accompanied by a concomitant increase in dry mass, which varies from 177 ± 11% to 320 ± 35% (Fig. 2A). While we observe that some cells escape from the composite and proliferate in the medium, this increase in dry mass suggests that most of the cells are retained in the hydrogel matrix. We also note that the shape of the grown composites is largely stable for over 128 days in deionized water at room temperature (fig. S4). The increase in dry mass and shape stability further supports our hypothesis that CO_2_ production or passive swelling from the hydrogel matrix is not the mechanism responsible for shape change. This mass change represents material that can be produced on demand with only as much external intervention or equipment as is needed to ferment grape juice. In the case of materials with 18 wt % yeast, the solid components of the as-synthesized composites are 35.9% polymer and 64.1% yeast. After growth, the yeast content increases to 85.6%. These growing composites may provide opportunities to produce materials directly from renewable feedstocks or even waste streams (*21*).

The mechanical properties of the hydrogel matrix also control the proliferation-induced shape change. By altering the feed ratio of cross-linker from 0.05 to 0.6% (w/v), at constant yeast loading (6 wt % dry yeast) and acrylamide concentration [10% (w/v)], the Young’s modulus of the composites after synthesis increases from 8 ± 1 kPa to 204 ± 16 kPa. As stiffness increases, the volume change during cell proliferation decreases from 255.8 ± 7.3% to 107.9 ± 1.2% (Fig. 2B and fig. S5). We attribute this decrease to increased elastic resistance to the expanding colonies, perhaps resulting in limited cell proliferation. Given the tradeoffs between composite stiffness, yeast loading, and volume change, we selected composites with 0.1% (w/v) cross-linker and 6 wt % yeast for further studies, as these composites have relatively high initial elastic modulus and large stimulus response (fig. S6).

Spatial control of volume change can be programmed to yield composites that morph controllably from 2D to 3D. Informed by previous work where spatially controlled swelling is used to guide shape selection in hydrogels (*22*–*26*), we fabricated composite disks (12 mm in diameter and 0.5 mm in thickness) and used UV light (254 nm) to kill cells in programmed areas (insets of Fig. 2, C and D). After irradiation, no shape change is observed when the hydrogel is equilibrated in water, indicating that the passive swelling of the gel is not substantially altered. After incubation in YPD, spatially controlled proliferation induces a 2D to 3D transformation. The flat disk shown in Fig. 2C grows in area in the center of the disk while being constrained around the perimeter, resulting in a hemispherical cap (+ Gaussian curvature). By contrast, the disk depicted in Fig. 2D grows along the perimeter while being constrained in the center, resulting in a saddle-like geometry (− Gaussian curvature).

**Fig. 3:**
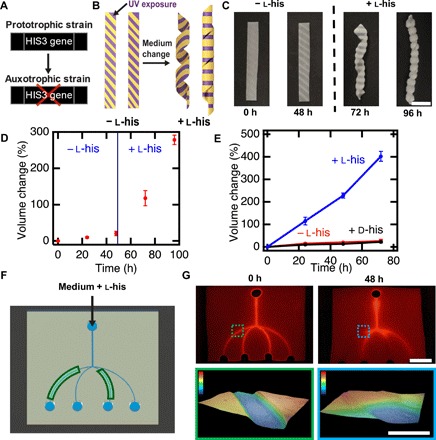
Genetic engineering enables controlled composite response to specific cues. (**A**)Deletion of the *HIS3* gene results in failure to proliferate in medium lacking histidine. (**B**) Schematic of a UV-patterned living composite with growth triggered by the amino acid l-histidine. (**C**) UV-patterned living composites with auxotrophic yeast do not substantially change in shape in medium lacking l-histidine. Shape change into a helical structure after incubation in medium containing l-histidine. Scale bar, 10 mm. (**D**) Volume change over time for auxotrophic living composites before and after l-histidine exposure. (**E**) Volume change over time for auxotrophic living composites incubated in medium lacking histidine, with d-histidine, or with l-histidine. (**F**) Schematic of a living microfluidic device where the composites forming the channels indicated in green contain living auxotrophic yeast. (**G**) Fluorescence image of fluid traversing the microfluidic device before exposure to medium (top left). Scale bar, 10 mm. Fluorescence image of fluid traversing the microfluidic device after medium containing l-histidine flows for 48 hours through the channels. Topography of a living channel before and after (color scale, 0 to 0.3 mm) growth (bottom). Scale bar, 1 mm. Each data point represents the mean (*n* = 3), and error bars represent SD. Trend lines are only intended to guide the eye.

Programming of the stimulus that induces shape change of living composites can be achieved by genetic manipulation of the yeast. *S. cerevisiae* is a model eukaryote commonly used for heterologous protein expression (*27*–*29*). The yeast strain we use (L40) is deficient in l-histidine metabolism. This metabolic feature, termed auxotrophy, prevents proliferation in the absence of l-histidine in the growth environment (Fig. 3A). This strain was used to fabricate composites that morph into 3D helical shapes only in the presence of l-histidine. Rectangular free-standing films were patterned with UV light to cause cell death in the areas indicated in Fig. 3B. Incubation for 48 hours in selective medium lacking l-histidine did not affect the shape of the composite. When these composites were incubated in medium containing l-histidine, the flat films morph into a helix (Fig. 3C). Over 48 hours in medium lacking l-histidine, disks of these composites only increase in volume by 20.7 ± 6.1%, despite the other 20 amino acids, d-glucose, and nitrogen base present in the medium. The same composites were then incubated for another 48 hours in otherwise identical medium containing l-histidine, resulting in a volume change of 278.3 ± 12.9% (Fig. 3D and fig. S7). Critically, these composites remain dormant during periods of unfavorable conditions and then respond when conditions match those programmed by the genetics of the yeast. The ability to withstand unfavorable conditions stands in stark contrast to the fragile nature of cells from multicellular organisms. To further demonstrate the biochemical specificity of these composites, volume change of living composites was measured for samples incubated in medium containing l-histidine and compared to the volume change of samples grown in medium with d-histidine and without any histidine. Much like the composites exposed to medium lacking l-histidine, the inclusion of d-histidine into the medium did not lead to substantial volume change at the end of an incubation period of 72 hours, as d-histidine is not naturally incorporated into proteins (Fig. 3E and fig. S7). Previously, hydrogels that swell by recognition of a single enantiomer of a chiral molecule have been achieved through molecular imprinting; however, the volume change in these materials is often less than 20% (*30*). Notably, the reported living composite exhibits a volumetric change up to 20× larger than the volume change observed in imprinted hydrogels.

Microfluidic devices fabricated with living composites respond with specificity to the fluid flowing through the channels. We fabricated responsive microfluidics by using replica molding to form channels within a living composite. All cells were rendered inviable with UV light except for the cells within two of the microfluidic channels (Fig. 3F). Flow of a fluorescent fluid is then used to visualize the performance of these devices. Before flowing medium containing l-histidine, the channels are all open, and the intensity of the fluorescence is similar across the device (Fig. 3G). After flowing medium with l-histidine through the inlet, the two microchannels with viable cells grow in volume, resulting in the channels becoming blocked, while the other channels remained open. By contrast, similar devices exposed to otherwise identical medium lacking l-histidine have all channels open (fig. S8). These smart microfluidic devices could enable strategies for biosensors that directly manipulate the flow of fluid without external intervention, traditional sensors, or actuators. While materials that respond to biochemical cues are ideal for devices that change autonomously with their environment, diffusion limits the ability of these cues to generate on-demand shape change with control in space and time.

**Fig. 4:**
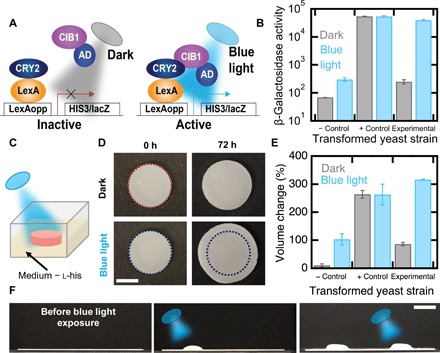
Genetic engineering enables optogenetic control of shape change. (**A**) Schematic of a light sensitive yeast two-hybrid. Blue light induces expression of *HIS3* and *lacZ* reporters by inducing conformational changes in CRY2 to favor interaction with CIB1. Reporter genes are transcribed by recruitment of the Gal4 activation domain (AD). (**B**) β-Galactosidase assays of an auxotrophic strain lacking CIB1 (negative control), a strain not auxotrophic for l-histidine in the dark (positive control), and the auxotrophic strain depicted in (A) (experimental). (**C**) Schematic of a living composite irradiated with blue light in growth medium lacking l-histidine. (**D**) Volume change of living composites with experimental yeast irradiated with blue light or kept in the dark. Scale bar, 5 mm. (**E**) Volume change of living composites with each yeast strain when exposed to blue light or kept in the dark. (**F**) Patterned photoresponsive living composite with the experimental yeast strain in medium lacking l-histidine where blue light is first targeted on the left side and then the right side. Scale bar, 10 mm. Each data point represents the mean (*n* = 3), and error bars represent SD.

Optogenetic switches can be engineered into yeast to enable photoresponsive composites, where shape change can be spatiotemporally controlled. We generated a yeast strain to express a photoresponsive transcriptional switch that induces gene expression after illumination with blue light (455 nm). This strain has two *Arabidopsis thaliana* proteins in a yeast two-hybrid system (*31*). Blue light stimulation induces binding of CRY2 fused to the LexA DNA binding domain and CIB1 Gal4 activation domain chimera. In the presence of light, the *HIS3* gene is activated, enabling cellular proliferation in the absence of l-histidine (Fig. 4A). We also generated two additional strains: a positive control that does not require light for activation of *HIS3* (*32*) and a negative control that lacks the CIB1 protein and is auxotrophic for l-histidine with or without blue light. The metabolic activity of the experimental strain is more than 100× higher when exposed to light than when kept in the dark, as measured by a β-galactosidase assay, which probes *lacZ*, a reporter gene, activity (Fig. 4B and fig. S9). In the negative control strain, the metabolic activity is low in blue light and in the dark, while the positive control presented high metabolic activity in both conditions.

Optogenetic control of histidine auxotrophy enables photocontrolled cellular proliferation and therefore shape change in living composites. We fabricated composites from each of the three different strains described above and exposed each composite to brief, dim pulses of blue light (2.7 mW/cm^2^, 2 s on, 2 min off) or darkness (Fig. 4C). Over 72 hours, living composites made with the experimental strain undergo a volume change of 315.9 ± 2.1% when exposed to light and a volume change of 86.2 ± 5.7% when kept in the dark (Fig. 4, D and E). By comparison, the positive control composites undergo a volume change of more than 260% in both light and dark, and negative control composites grow only 103.1 ± 20.1% in the light and 10.2 ± 5.3% in the dark (Fig. 4E and fig. S9). We note that traditional photoresponsive polymers, which use light to power shape change, typically require irradiation intensities of more than 100 mW/cm^2^ (*33*, *34*). By comparison, these living composites rely on light to trigger an optogenetic switch that has been optimized by evolution. The activation of this switch subsequently enables a metabolically powered change in volume. As a result of this pathway, the time-averaged intensity required is at least 2250× smaller than traditional photoresponsive polymers.

The combination of patterned cell viability and patterned light illumination can be used to provide spatiotemporal control of complex shape change in living composites composed of transformed yeast with the optogenetic switch. The cell viability within a film of the living composite was patterned using UV light to leave only two circular regions on the film with viable cells (fig. S10). As shown in Fig. 2C, proliferation should lead to the formation of a hemispherical cap. By exposing these two regions sequentially, each region sequentially actuates from flat to hemispherical (Fig. 4F).

Living composites undergo cell proliferation–induced shape change controlled by the initial composition of the composite or by patterning regions of viable cells. These materials capitalize genetic control of biological mechanisms, namely, cellular proliferation, to enable responsiveness in topography or shape in response to specific cues. A host of devices from drug delivery platforms to environmental sensors could be enabled by these findings.

## MATERIALS AND METHODS

### Materials

Acrylamide, *N*,*N*′-methylenebisacrylamide (MBAA), ammonium persulfate (APS), *N*,*N*,*N*′,*N*′-tetramethylethylenediamine (TEMED), 3-amino-1,2,4-triazole (3-AT), l-histidine, adenine sulfate, sulforhodamine B, bisphenol A ethoxylate diacrylate (BPA) (512 g/mol), and poly(ethylene glycol) diacrylate (PEG-DA) (700 g/mol) were purchased from Sigma-Aldrich. The photoinitiator Irgacure 369 (I-369) was donated by BASF Corporation. Methacryloxyethyl thiocarbonyl rhodamine B (PolyFluor 570) was purchased from Polysciences. Commercial yeast (*S. cerevisiae*, active dry yeast, Fleischmann’s) was purchased from Tom Thumb (Richardson, TX). Yeast extract, yeast nitrogen base without amino acids, peptone, d-(+)-glucose, d-histidine, and trypan blue were purchased from Fisher Scientific. TPM [3-(trimethoxysilyl) propyl methacrylate] was purchased from Acros Organics. Rain-X was purchased from Wal-Mart (Richardson, TX). All chemicals were used as received without further purification.

### Genetically engineered yeast strains and plasmids

The genotype of the L40 yeast strain is *MATa ade2 his3 leu2 trp1 LYS::lexA-HIS3 URA3::lexA-LacZ* (American Type Culture Collection, MYA-3332) (*35*). L40 yeast were transformed with experimental constructs CRY2 LexA DNA binding fusion in the expression vector pDBTrp (pDBTrp-LexABD-CRY2FL) (plasmid no. 78210, Addgene) and a separate CIB1 Gal4 activation domain fusion vector pGADT7 (pGal4AD-CIB1) (plasmid no. 28245, Addgene). pDBTrp-LexABD-CRY2FL, along with the pGADT7 empty vector, was used as negative control. In the positive control, we made use of a previously described interaction between *Caenorhabditis elegans* FBF2 (residues 121 to C terminus fused to the Gal4 activation domain present in pGADT7) and CPB1 (residues 1 to 80 fused to the LexA DNA binding domain encoded by pBTM116) (*32*, *36*).

### Mold construction

For volume change and mechanical testing experiments, molds were made of two glass slides (75 mm by 51 mm) previously cleaned with Rain-X to avoid gel adhesion. Slides were separated with 1-mm or 500-μm rectangular spacers, wrapped with parafilm closing one of the open sides, and fixed using binder clips.

For living composite coatings, molds (75 mm by 25 mm) with one glass slide cleaned with Rain-X and one treated with a methacrylate-functionalized silane were assembled. The two glass slides were separated with two 250-μm polystyrene spacers on each side and fixed with binder clips. For the silane treatment, glass slides were cleaned following a similar process described in the literature (*37*). Briefly, glass slides were sonicated for 5 min in acetone and isopropanol mixtures and rinsed three times in dH_2_O. Afterward, substrates were sonicated for 30 min in a mixture of water and Alconox cleaner (Alconox Inc., USA), rinsed, and stored in dH_2_O overnight. For silanization, glass slides were modified for 30 min with a 5% (v/v) mixture of TPM in toluene at 65°C. Then, the slides were rinsed with toluene, dried with N_2_ gas, and baked on a hot plate at 120°C for 5 min.

### Determination of cell density

To determine cell concentrations in active dried yeast, cell density was measured with a UV/visible spectrophotometer by observing the optical density at 660 nm. Briefly, 50 ml of mixture with 0.6 g of yeast in dH_2_O was prepared. Then, a 1:10 dilution was made by mixing 0.1 ml of the mixture with 0.9 ml of dH_2_O. Diluted samples were pipetted into a 1-ml cuvette for spectrophotometer measurements. Optical densities between 1 and 1.1 were measured, which correspond to numbers of cells of 1.89 × 10^7^ and 2.25 × 10^7^ cells, respectively. These results indicate that the active dried yeast contained between 15 billion and 18 billion cells/g.

### Preparation of living composite materials with active dried yeast

Polyacrylamide hydrogels with embedded yeast were prepared at room temperature by free radical polymerization of acrylamide monomer and MBAA cross-linker. Stock solutions of acrylamide (0.4 g/ml) and MBAA (0.02 g/ml) were prepared in dH_2_O to create polyacrylamide gel precursor solutions. All pre-gel solutions were prepared with a final concentration of 10% (w/v) acrylamide. Pre-gel solutions were prepared with a final concentration of 0.1% (w/v) MBAA and pre-gel solution (~1 billion cells/ml), unless otherwise noted. To polymerize these solutions, a 10% (w/v) APS stock solution was added at 1% of the total solution volume, and TEMED was added at a ratio of 0.1% of total solution volume. Polymerizing solutions were then vortexed for 3 s and quickly pipetted into molds. Filled molds were flipped every 45 s, while polymerization occurred to avoid yeast settling. After 10 min, polymerized living composites were demolded and rinsed three times with dH_2_O to remove unpolymerized acrylamide residues. Living composites were stored in dH_2_O for 24 hours before mechanical testing and volume change experiments. For mechanical testing and volume change experiments, pre-gel solutions were prepared with 0.05, 0.1, 0.3, and 0.6% (w/v) MBAA and 6% (w/v) yeast [pre-gel solution (~1 billion cells/ml)]. To test volume change and Young’s modulus with varying yeast content, composites with final concentrations of 0, 1, 6, 12, and 18 wt % yeast were prepared.

### Area, volume, and mass change quantification of living composites embedding active dried yeast

Living composites embedded with commercial active dried yeast were cut into 10-mm-diameter disks. The dimensions of each disk were measured before incubation. Samples with varying dry yeast and cross-linker concentration were incubated at 30°C in YPD-rich medium without agitation (1% yeast extract, 2% peptone, and 2% d-glucose). To measure area and volume changes, three samples for each composition were incubated in 7 ml of rich medium that was changed every 6 hours. Area change was measured every hour for 48 hours using a Mighty Scope 5M digital microscope, and volume changes were measured every 24 hours for 48 hours using a Canon Rebel T5i camera.

Dry mass change was obtained by weighing samples with varying yeast content before and after cell proliferation. Briefly, one set of living composites that was not exposed to medium was dried at 30°C under vacuum to allow water evaporation. An identical set of living composites was incubated in YPD medium for 48 hours with medium change every 6 hours and then dried under the same conditions. Upon drying, samples were weighed and mass was measured. Data presented are an average of three samples per composition.

### Material characterization

Samples (3 mm by 3 mm by 1 mm) were cut from polymerized living composites with varying yeast and cross-linker content after equilibration in dH_2_O. Compression testing was performed using a MicroSquisher (CellScale Biomaterials Testing). Briefly, a tungsten beam with a diameter of 1.016 mm was glued to a 6 mm–by–6 mm platen on one end. This compliant beam was attached to an actuator with a cantilever beam grip at the opposite end from the platen. Samples were loaded to the test chamber filled with dH_2_O at room temperature. The beam was brought into contact with the sample and then moved at a rate of 0.5 mm/min. Force as a function of displacement was measured along the height (1-mm dimension) of the samples and calculated by the MicroSquisher software using the beam’s stiffness, displacement, and length. Strains from 1 to 10% were used to calculate Young’s modulus, as the stress-strain response in this region was linear.

### Optical images of living composites

Microscopic imaging was carried out using an Olympus FV3000RS confocal laser scanning microscope. To visualize embedded yeast cell budding, living composites with 10% (w/v) acrylamide and 0.1% (w/v) MBAA were synthesized, mixing approximately 1 × 10^6^ cells/ml of the pre-gel solution. Before polymerization, a 0.05% (w/v) aqueous solution of PolyFluor 570 was added at 1% of the total pre-gel solution volume. Cells, after incubation for 48 hours in YPD medium, were further stained by submerging samples in a 0.05% (w/v) aqueous solution of trypan blue for 3 min and then washed two times in dH_2_O. Budding of embedded cells and colonies were observed throughout the thickness and area of the imaged samples (*n* = 3).

### Macroscopic fluorescence images

For fluorescence imaging, living composites were dyed with a 0.05% (w/v) solution of sulforhodamine B in water. By shining light at a wavelength of 455 nm, fluorescent images of the UV-patterned coatings and free-standing structures were obtained using a DSLR camera (Canon Rebel T5i) fitted with a red filter (Hoya HMC R25A). This filter blocks light below 600 nm, thus allowing visualization of the emitted light.

### UV photopatterning of composites with active dried yeast

Living composites covalently bound to methacrylate-functionalized glass molds with a thickness of 500 μm were prepared as described above. Composites were allowed to equilibrate in water before UV exposure. A shadow mask of the word ALIVE was designed in AutoCAD and laser cut from black polymer sheets. Irradiation with 254-nm UV light with an intensity of 2 mW/cm^2^ was performed from one side for 35 min using an UVP UVLink 1000 cross-linker chamber. Samples were placed on a dark background during irradiation.

For UV patterning of free-standing composites, 12-mm-diameter disks were cut from 500-μm-thick films and patterned to induce cell death in a 6-mm-diameter inner circle or a 3-mm-wide ring pattern using aluminum foil as a mask. Irradiation with 254-nm UV light with an intensity of 2 mW/cm^2^ was performed from one side for 35 min using the UV chamber.

For cylindrical helix patterning, living composite samples were synthesized with the same composition of monomers as described above and a pre-gel solution (0.9 billion to 1.1 billion cells/ml). Samples were cut into rectangular shapes (length, 40 mm; height, 5 mm; and thickness, 0.5 mm) and patterned to induce cell death in 2.3-mm-wide rectangles separated by 1.1 mm and positioned at 56° angle along the length of the samples (Fig. 3A). Irradiation was performed with the same wavelength, intensity, and time as described above.

After irradiation, living composites bound to glass were incubated for 36 hours at 30°C with a medium change every 12 hours. Free-standing disks were incubated for 48 hours at 30°C with medium change every 12 hours. These samples were then imaged using a fluorescent dye as described above. Cylindrical helix films were incubated for 48 hours at room temperature with medium change every 6 hours and then imaged using a Mighty Scope 5M digital microscope every 5 min.

### Topography measurements of UV photopatterned living composite coatings

Topography (Fig. 1F) of living composite coatings was imaged using a digital microscope (Keyence VHX-1000). To characterize the change in film thickness after cell proliferation, measurements along the depth profile of the letter “A” were taken. The camera limit points were set by focusing on the highest point of the grown letter and on the coated UV-killed surface. Between these limits, images were taken at ×100 magnification along the surface of the letter. Images were then stitched using the KEYENCE software.

### Quantifying shape change in living composites with auxotrophic yeast strain

Before composite synthesis, the auxotrophic yeast strain (CRY with empty vector, denoted as negative control strain) was grown overnight in selective medium [0.7% yeast nitrogen base without amino acids, 2% d-glucose, and appropriate amino acid supplements (*38*) lacking tryptophan, leucine, and histidine] containing l-histidine. Subsequently, overgrowths were made in 50 ml of YPAD (1% yeast extract, 2% peptone, 0.004% adenine sulfate, and 2% d-glucose) medium at 30°C for 15 hours. Growth was followed by measuring optical density at 660 nm until desired yeast concentration was reached (OD_660_ = 1 to 1.1). Cells were then centrifuged in 50-ml conical tubes and washed twice in distilled water before encapsulation. Composites with encapsulated auxotrophic yeast were synthesized by using 10% (w/v) acrylamide and 0.1% (w/v) MBAA with pre-gel solution (0.9 billion to 1.1 billion cells/ml), as described above. For volume change experiments, living composites were first equilibrated in water for 24 hours and then cut into 10-mm-diameter disks with a thickness of 500 μm. Disks were incubated in selective medium lacking l-histidine with 10 mM 3-AT (*HIS3* gene competitive inhibitor) for 48 hours at 30°C, with a medium change every 12 hours. After this time, composites were incubated in selective medium containing l-histidine and 10 mM 3-AT for another 48 hours, with a medium change every 12 hours. Disks were measured after growth for quantification of volume change.

Identical disks were also exposed to selective medium containing l-histidine, selective medium containing d-histidine, a stereoisomer of the natural amino acid l-histidine, and selective medium without l-histidine. These composites were incubated for 72 hours at 30°C, with a medium change every 12 hours. Data presented are an average of three samples per experiment.

### Controlled blockage of microfluidic device

For shape change experiments using a microfluidic device, composites were cast into microfluidic polymer micromolds. These molds were built with 75 mm–by–51 mm glass slides. One of the two slides was functionalized with methacrylate groups by the processes described above. The other slide was coated in Rain-X. Slides were separated with one 250-μm polystyrene spacer on each side, and BPA mixture with 1 wt % I-369 photoinitiator was pipetted into the mold. Using a Vivitek D912HD (B9Creator) projector with the UV filter removed and the optics modified to decrease the focal length, a positive mold of a microfluidic device with a 600-μm-wide inlet channel and four 400-μm-wide outlet channels was polymerized onto the methacrylate-functionalized glass slide. To remove all unpolymerized BPA, slides were cleaned by multiple washes between acetone and isopropanol. This micromold was then used to create a cell for the polymerization of the living composite. After polymerization, microfluidic devices were allowed to equilibrate in dH_2_O for 24 hours. Using UV patterning (254-nm wavelength, 35 min, 2 mW/cm^2^ intensity), most of the composite was rendered inviable. Two of the four outlet channels, as indicated in Fig. 3F, were kept alive by preventing UV exposure on the channel areas with a shadow mask. Selective medium containing l-histidine was then flowed at a rate of 34 μl/min through one set of microfluidic devices (*n* = 3), and selective medium without l-histidine was flowed through another set of devices (*n* = 3) at the same rate. Before and after medium flow, channels were injected with a PEG-DA solution mixed with sulforhodamine B aqueous solution at 1% of the total PEG-DA volume. Flowing this solution allowed visualization of the flow through the microchannels before and after growth. Topographical quantification of these samples was performed following the same process described for living composite coatings.

### Yeast two-hybrid assays

L40 cells were cotransformed with the appropriate plasmids as described above and in the literature (*39*). Cells were plated on selective medium agar plates with 10 mM 3-AT. Two replicate plates were grown in the dark, and two were irradiated with blue light at 455 nm for 2 s every 2 min. Plates were incubated at 30°C for 3 days.

Transformants were grown to saturation in selective medium lacking l-histidine overnight at 30°C. Afterward, aliquots (100 μl) of the saturated cultures were outgrown in 1 ml of fresh minimal medium and incubated at 30°C for 4 hours. Optical density at 660 nm was recorded using a Spark 20 M multimode reader. β-Galactosidase expression was quantified using the Beta-Glo Assay System (Promega). Briefly, 50 μl of cell cultures was added to an equal volume of reagent and allowed to incubate for 45 min before quantification. The luminescence values were normalized to cell densities for each culture.

### Optogenetic control of shape change

Control of proliferation by exposure to blue light was achieved by encapsulating experimental yeast strains that express photosensory proteins. Pre-gel solutions with a composition of 10% (w/v) acrylamide and 0.1% (w/v) cross-linker were mixed with 0.9 billion to 1.1 billion transformed cells per milliliter of solution. Positive and negative control strains were encapsulated using the same pre-gel composition and cell concentration. For volume change experiments, composites with each of the three strains were cut into 10-mm-diameter disks with a thickness of 500 μm. Samples were incubated in selective medium lacking l-histidine with 10 mM 3-AT for 72 hours with medium change every 12 hours. Then, samples were irradiated with blue light (455 nm) (Fig. 4C) or kept in the dark. The intensity of irradiation was measured with a solar power meter (Amprobe SOLAR-100) and set at 2.7 mW/cm^2^. For blue light experiments, samples were irradiated for 2 s every 2 min. Volume changes were measured every 24 hours for all samples before and after incubation. Data presented are an average of three samples per experiment.

For spatiotemporal control of proliferation, free-standing films with encapsulated experimental yeast strain were UV-patterned as shown in fig. S10. Films were kept in dH_2_O for 24 hours in the dark before blue light exposure. Films were then incubated in selective medium with 10 mM 3-AT at 30°C in the dark for 72 hours. For the first 48 hours, only the left half of the film was exposed to blue light to induce proliferation of cells. After this time, the right half was irradiated for 24 hours. Images are representative from three trials.

## Supplementary Material

http://advances.sciencemag.org/cgi/content/full/6/3/eaax8582/DC1

Download PDF

Movie S1

Movie S2

Shape-morphing living composites

## SUPPLEMENTARY MATERIALS

Supplementary material for this article is available at http://advances.sciencemag.org/cgi/content/full/6/3/eaax8582/DC1 Fig. S1. Change in area depends on initial cell loading. Fig. S2. Representative images of the living composites before and after incubation in medium. Fig. S3. Buckling pattern in living composite coated on a glass substrate. Fig. S4. Shape change stability. Fig. S5. Representative images of the macroscopic expansion of living composites with varying cross-linker density. Fig. S6. Characterization of living composites with varying yeast content. Fig. S7. Living composite shape change induced by adding a specific biochemical. Fig. S8. Microfluidic device exposed to medium without l-histidine. Fig. S9. Yeast proliferation on minimal agar medium. Fig. S10. Optogenetic control of shape change in genetically engineered living composites. Movie S1. Volume change over time of a living composite with 6 wt % embedded yeast. Movie S2. Shape change of a living composite into a helical structure.
