# Supplementary material for: Shape-morphing living composites
Source: Sci Adv. 2020 Jan 17;6(3):eaax8582. doi: 10.1126/sciadv.aax8582 (PMC6968942; doi:10.1126/sciadv.aax8582)
Supplement: http://advances.sciencemag.org/cgi/content/full/6/3/eaax8582/DC1 [file supp_6_3_eaax8582__index.html]

Science Advances | Science AdvancesAAASSearchScience AdvancesMenu

## Supplementary Materials

**The PDFset includes:**

- Fig. S1. Change in area depends on initial cell loading.
- Fig. S2. Representative images of the living composites before and after incubation in medium.
- Fig. S3. Buckling pattern in living composite coated on a glass substrate.
- Fig. S4. Shape change stability.
- Fig. S5. Representative images of the macroscopic expansion of living composites with varying cross-linker density.
- Fig. S6. Characterization of living composites with varying yeast content.
- Fig. S7. Living composite shape change induced by adding a specific biochemical.
- Fig. S8. Microfluidic device exposed to medium without l-histidine.
- Fig. S9. Yeast proliferation on minimal agar medium.
- Fig. S10. Optogenetic control of shape change in genetically engineered living composites.
- Legends for movies S1 and S2

Download PDF

**Other Supplementary Material for this manuscript includes the following:**

- Movie S1 (.mp4 format). Volume change over time of a living composite with 6 wt % embedded yeast.
- Movie S2 (.mp4 format). Shape change of a living composite into a helical structure.

**Files in this Data Supplement:**

- Adobe PDF - aax8582\_SM.pdf
