# Supplementary material for: Shape-morphing living composites
Source: Sci Adv. 2020 Jan 17;6(3):eaax8582. doi: 10.1126/sciadv.aax8582 (PMC6968942; doi:10.1126/sciadv.aax8582)
Supplement: Download PDF [file aax8582_SM.pdf]

Science Advances
AAAS

advances.sciencemag.org/cgi/content/full/6/3/eaax8582/DC1

Supplementary Materials for

Shape-morphing living composites

L. K. Rivera-Tarazona, V. D. Bhat, H. Kim, Z. T. Campbell, T. H. Ware*

*Corresponding author. Email: taylor.ware@utdallas.edu

Published 17 January 2020, *Sci. Adv.* **6**, eaax8582 (2020)
DOI: 10.1126/sciadv.aax8582

The PDF file includes:

Fig. S1. Change in area depends on initial cell loading.
Fig. S2. Representative images of the living composites before and after incubation in medium.
Fig. S3. Buckling pattern in living composite coated on a glass substrate.
Fig. S4. Shape change stability.
Fig. S5. Representative images of the macroscopic expansion of living composites with varying cross-linker density.
Fig. S6. Characterization of living composites with varying yeast content.
Fig. S7. Living composite shape change induced by adding a specific biochemical.
Fig. S8. Microfluidic device exposed to medium without L-histidine.
Fig. S9. Yeast proliferation on minimal agar medium.
Fig. S10. Optogenetic control of shape change in genetically engineered living composites.
Legends for movies S1 and S2

Other Supplementary Material for this manuscript includes the following:

(available at advances.sciencemag.org/cgi/content/full/6/3/eaax8582/DC1)

Movie S1 (.mp4 format). Volume change over time of a living composite with 6 wt % embedded yeast.
Movie S2 (.mp4 format). Shape change of a living composite into a helical structure.

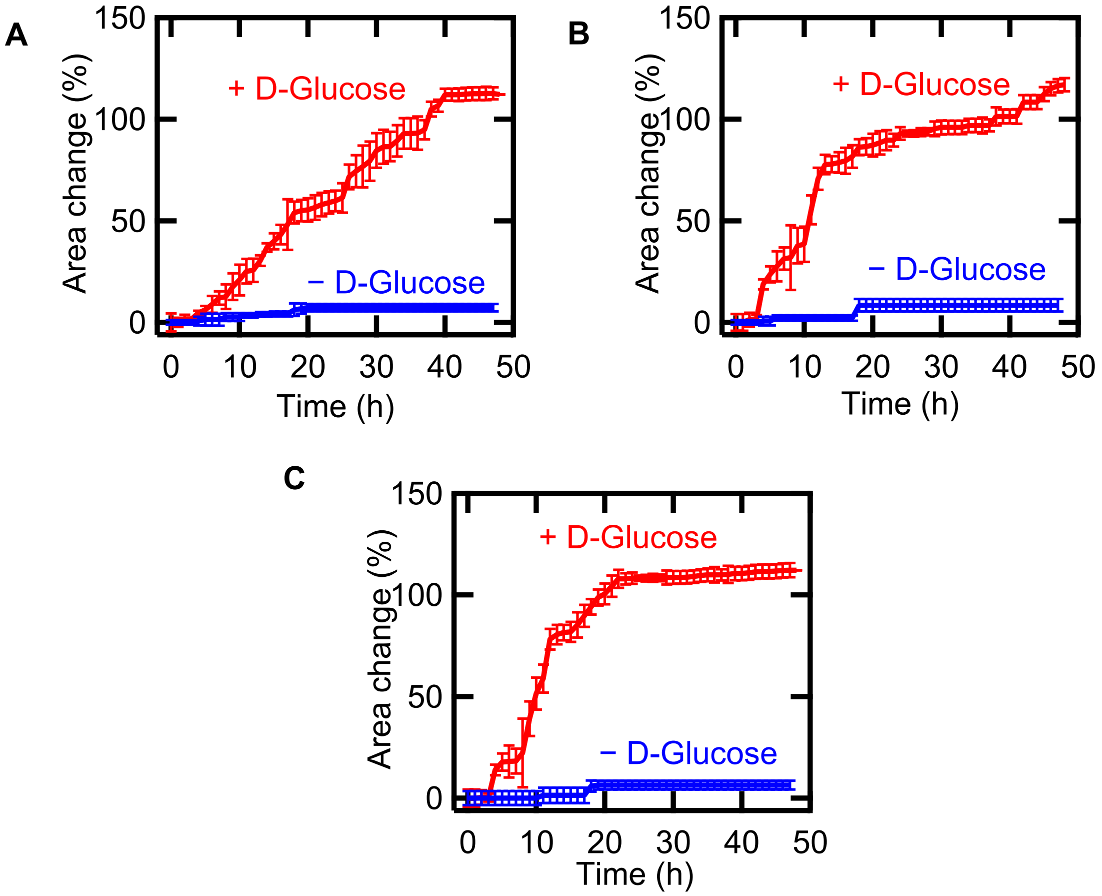

Fig. S1. Change in area depends on initial cell loading. Area change over time for composites with 1 wt% (**A**), 12 wt% (**B**) and 18 wt% (**C**) dry yeast in the presence of media with and without D-glucose. Each data point represents the mean (n=3), and error bars represent standard deviation. Trend lines are only intended to guide the eye.

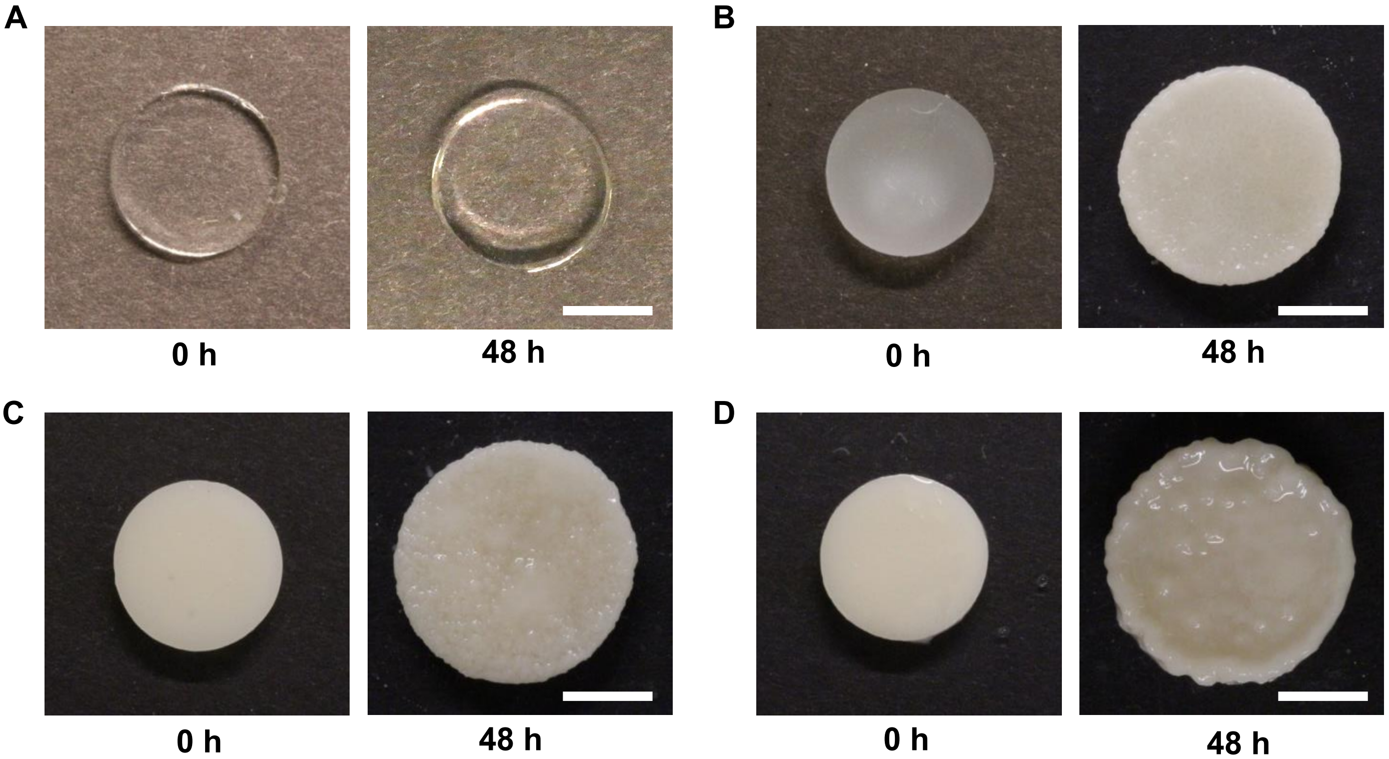

Fig. S2. Representative images of the living composites before and after incubation in medium. Images of hydrogels without yeast (**A**) and composites with 1 wt% yeast (**B**), with 12 wt% yeast (**C**), and 18 wt% yeast (**D**) (Scale bars: 5 mm). Each experiment had 3 replicates. All samples were incubated at 30ºC in YPD media under static conditions with media changed every 6 h.

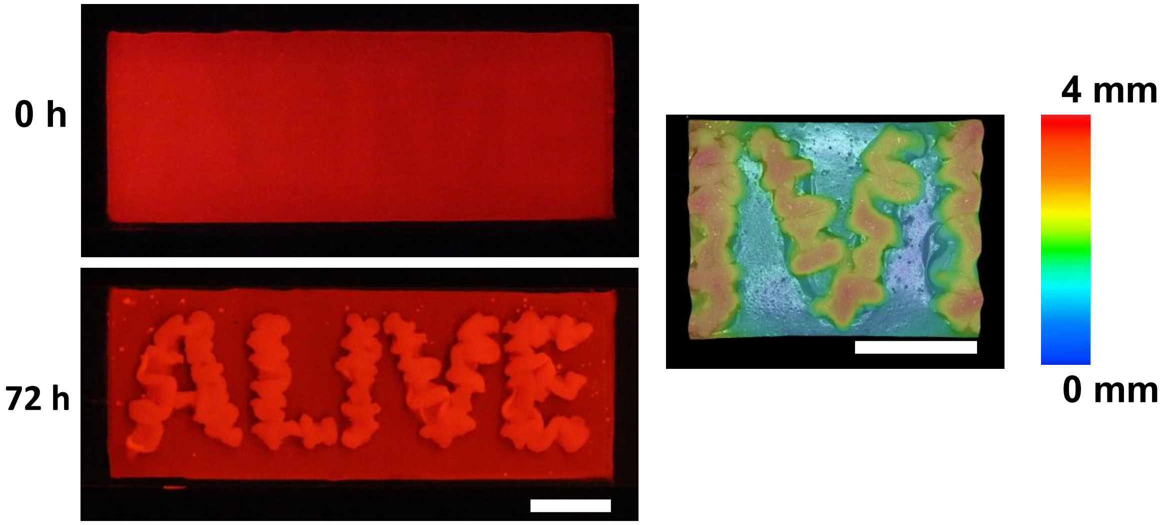

Fig. S3. Buckling pattern in living composite coated on a glass substrate. Fluorescence images of a living composite after UV patterning (top) and after incubation in YPD (bottom) (Scale bar: 10 mm). Topography of an initially flat living composite after exposure to YPD (right) (Scale bar: 10 mm). Samples were incubated at 30ºC in YPD media under static conditions with media changed every 12 h.

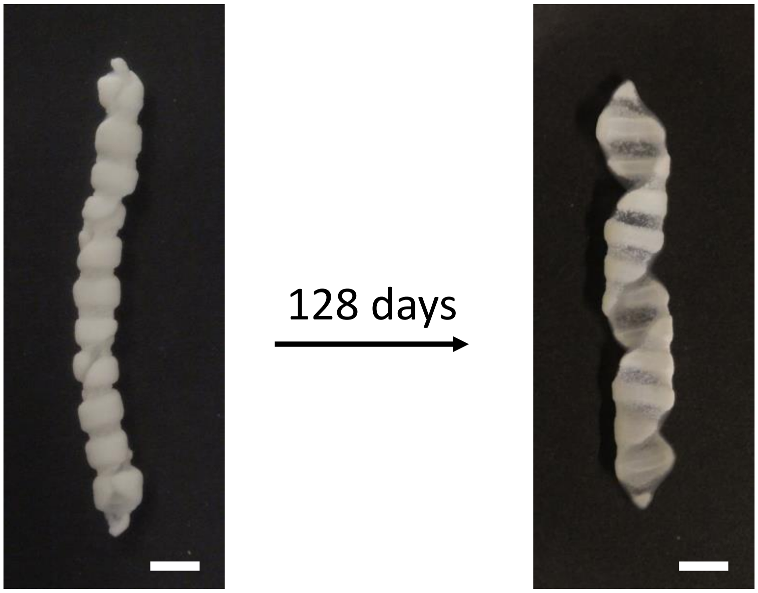

Fig. S4. Shape change stability. Helical structure after growth (left) and after storage for 128 days. Samples were stored in dH_2O at room temperature (Scale bars: 5 mm). Shape change due to proliferation is largely stable.

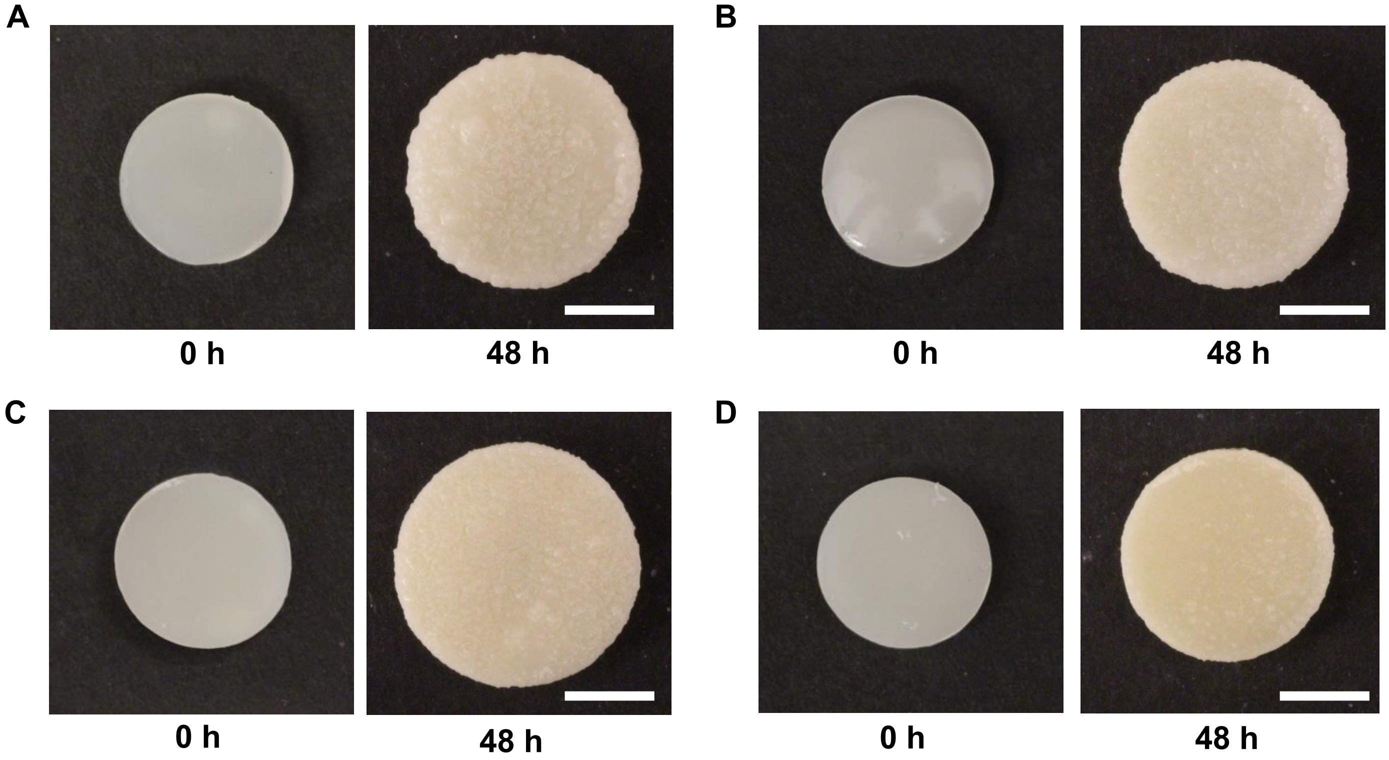

Fig. S5. Representative images of the macroscopic expansion of living composites with varying cross-linker density. Images of living composites synthesized with 0.05 wt% crosslinker (**A**), 0.1 wt% crosslinker (**B**), 0.3 wt% crosslinker (**C**), and 0.6 wt% crosslinker (**D**) (Scale bars: 5 mm). Each experiment had 3 replicates. All samples were incubated at 30ºC in YPD media under static conditions with media changed every 6 h.

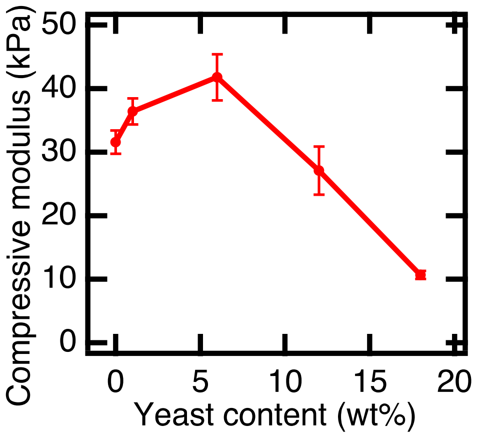

Fig. S6. Characterization of living composites with varying yeast content. Compressive modulus of living composites with varying yeast content. Each data point represents the mean (n=3), and error bars represent standard deviation. Trend lines are only intended to guide the eye.

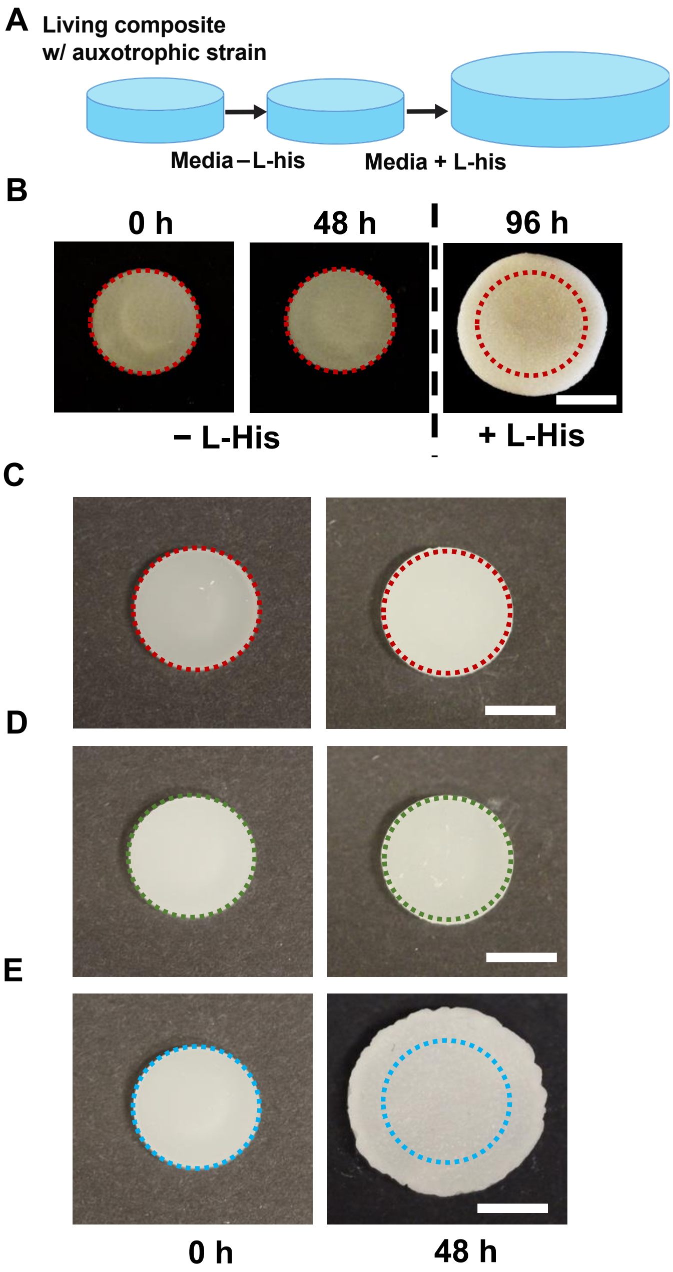

Fig. S7. Living composite shape change induced by adding a specific biochemical. (**A**) Schematic of a genetically modified living composite with growth triggered by the addition of L-histidine (**B**) Composites do not change in volume substantially in media lacking L-histidine but grow after L-histidine is supplemented into the media (Scale bar: 5 mm). Representative images of the macroscopic expansion of auxotrophic living composites incubated in media without histidine (**A**), with the stereoisomer D-histidine (**B**), and with L-histidine (**C**) (Scale bars: 5 mm). All samples were incubated at 30°C under static conditions and media was changed every 12 h. Dotted circles represent initial area and are used to guide the eye after composites growth.

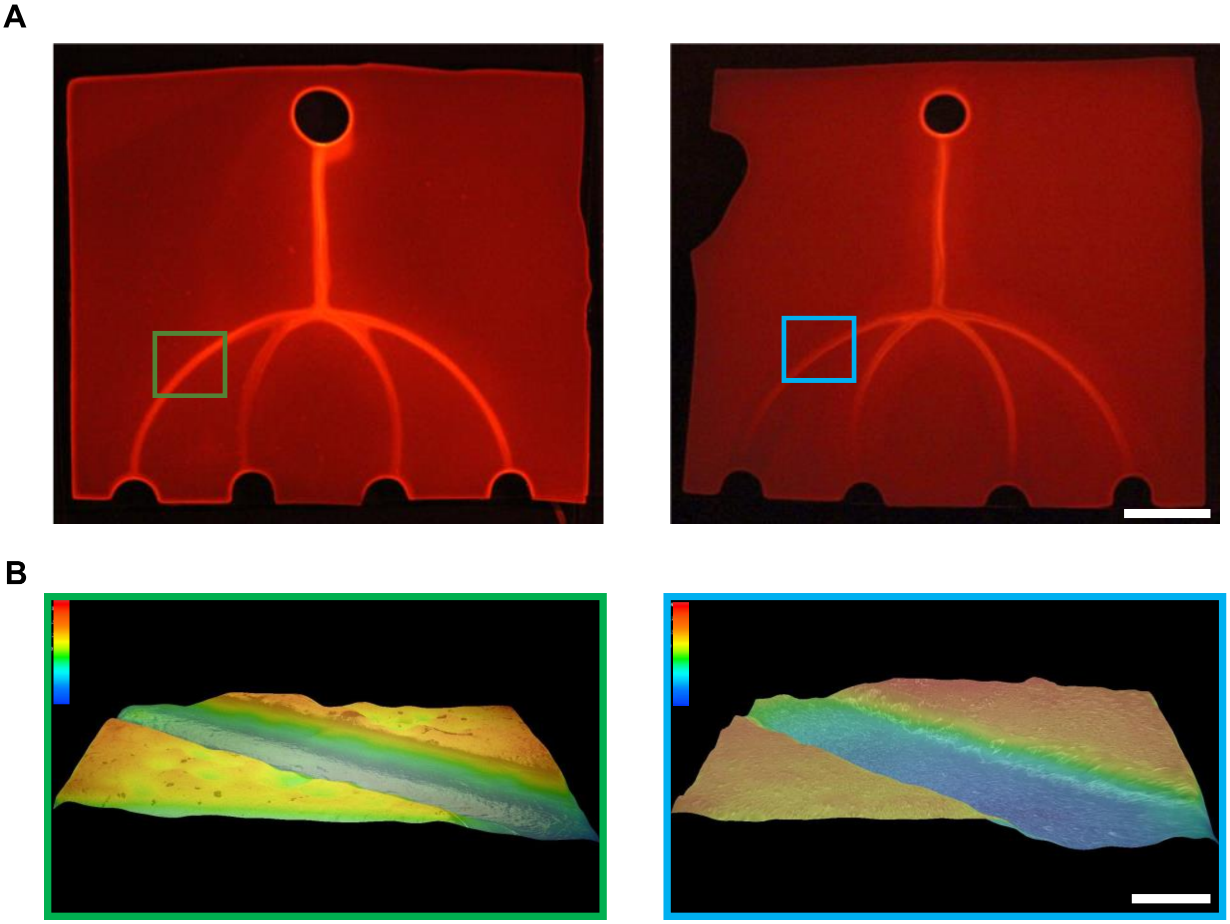

Fig. S8. Microfluidic device exposed to medium without L-histidine. (**A**) Fluorescence image of fluid traversing the microfluidic device before exposure to media (left) (Scale bar 10 mm). Fluorescence image of fluid traversing the microfluidic device after media lacking L-histidine flows for 48 h through the channels. Growth of areas with living cells is not affected by media flow (right). (**B**) Topography of a living channel recorded before (left) and after (right) growth (Scale bar: 0.5 mm) (Color scale: 0-0.3 mm).

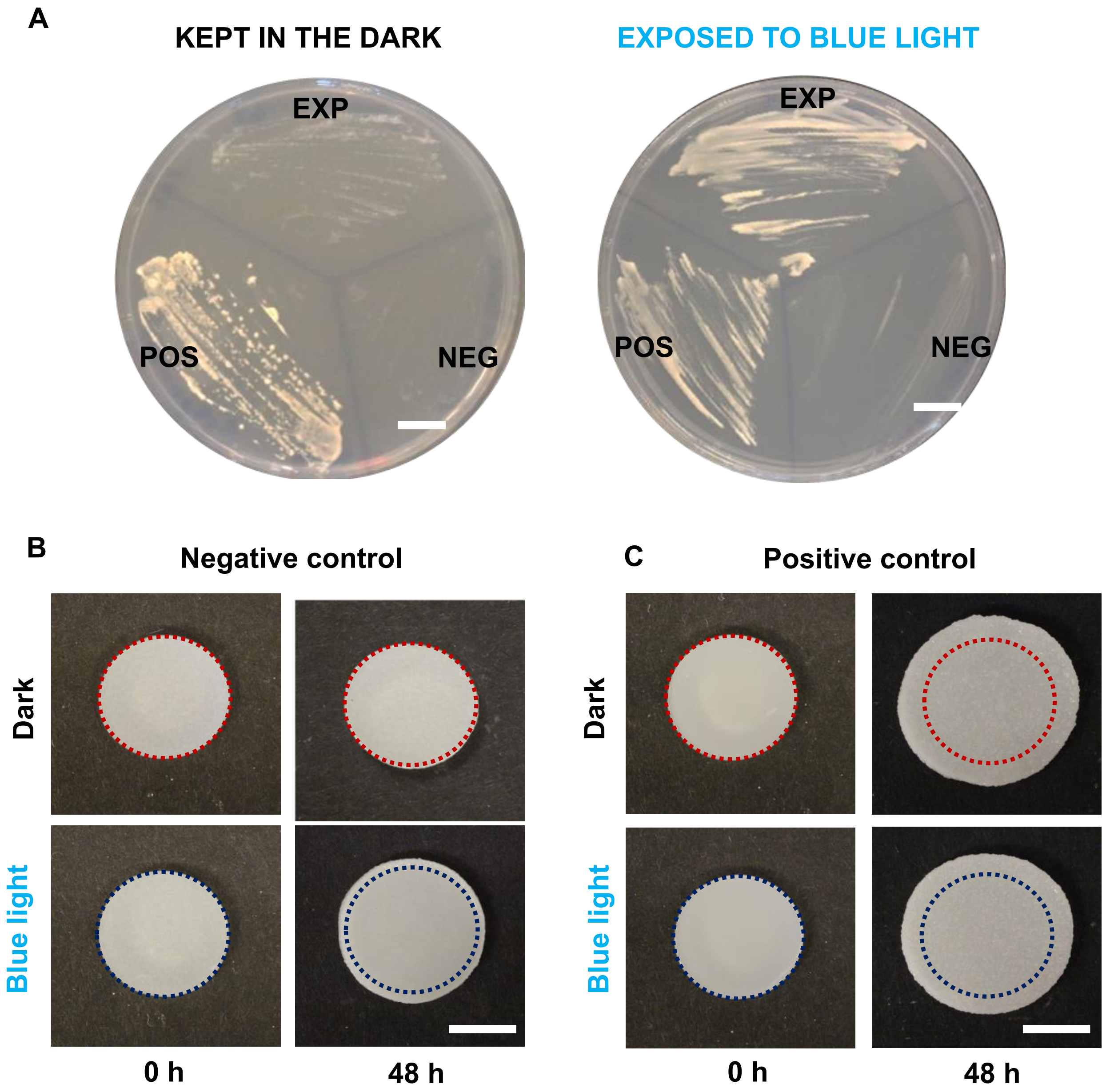

Fig. S9. Yeast proliferation on minimal agar medium. (**A**)Yeast strains growing on minimal SD agar without L-Histidine and with competitive inhibitor 3AT. Plates were incubated at 30ºC for 36 h. The plate on the left was wrapped in aluminum foil to avoid light exposure, and the plate on the right was exposed to blue light at 455 nm of wavelength with 2.7 mW/cm^2 2 s pulses every 2 min (Scales bars: 10 mm). POS refers to the positive control strain. NEG refers to the negative control strain. EXP refers to the experimental strain. Representative images of living composites kept in the dark or exposed to blue light. Negative control composites are shown in (**B**) and positive control composites in (**C**) (Scale bars: 5 mm). Composites are incubated at 30ºC in media without L-Histidine for 48 h with media change every 12 h. Composites kept in the dark are wrapped in aluminum foil and light exposed composites are irradiated with 455 nm wavelength light with intensity of 2.7 mW/cm^2 for 2 s every 2 min. Dotted circles represent initial area and are used to guide the eye after composites growth.

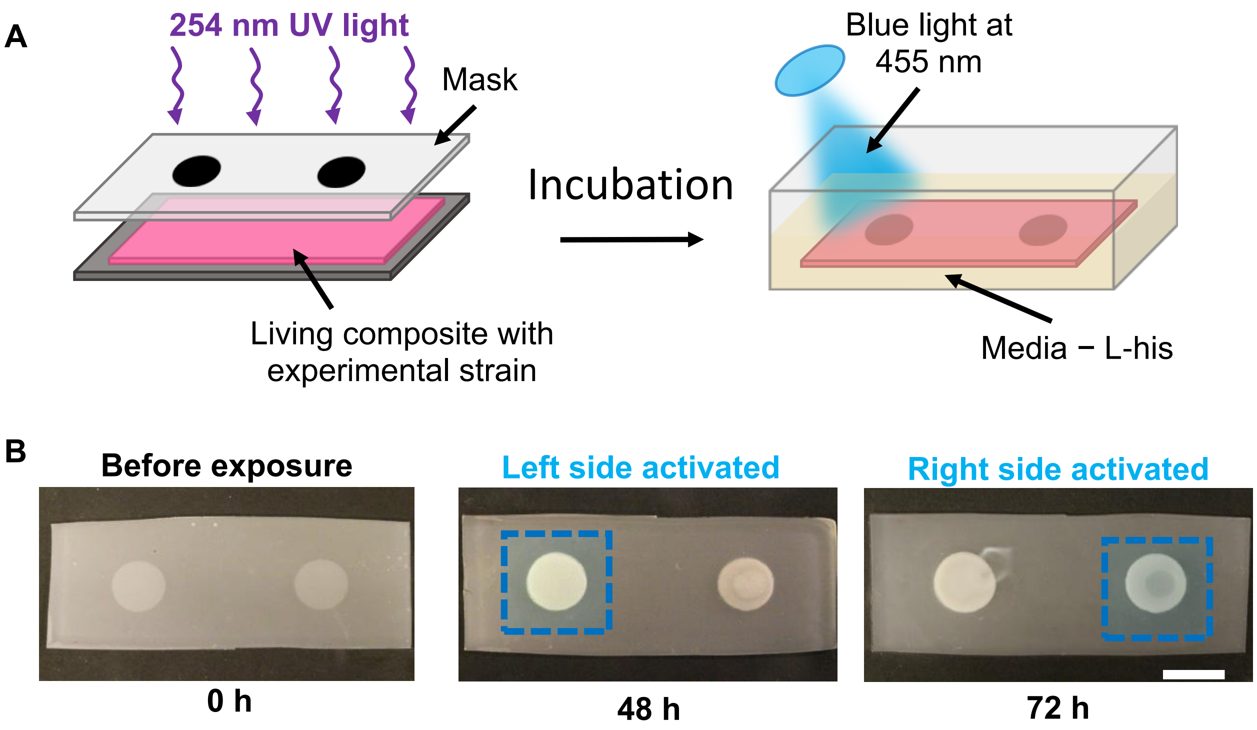

Fig. S10. Optogenetic control of shape change in genetically engineered living composites. (**A**) Schematic of the UV patterning of a free-standing film. Area indicated by the two circles contain living yeast while the area surrounding these circles contain cells that are UV-killed. After patterning, the living composite is equilibrated in dH_2O and incubated in media without L Histidine. During incubation, the living composite is irradiated with 455 nm wavelength blue light with an intensity of 2.7 mW/cm^2 for 2 s every 2 min over a portion of the sample. (**B**) Patterned composites before exposure to blue light (left), after incubation in media without L-histidine and only irradiating the left circle (middle), and irradiation of right circle only (right) (Scale bar: 10 mm).

Movie S1. Volume change over time of a living composite with 6 wt % embedded yeast. Time lapse video of a disk (10 mm diameter, 1 mm thickness) growing in YPD media at 30 °C. The sample was incubated for 48 h with a media change every 6 h. Images were taken every 5 min. The timelapse images are played at 10800x actual speed.

Movie S2. Shape change of a living composite into a helical structure. Time lapse video of a 40 mm x 5 mm x 0.5 mm free-standing film UV patterned as indicated in Figure 3B. Film was grown in YPD media at room temperature for 48 h with a media change every 6 h. Images were taken every 5 min. The timelapse images are played at 10800x actual speed.
